# Influence of Supplementation of Vegetable Oil Blends on Omega-3 Fatty Acid Production in* Mortierella alpina* CFR-GV15

**DOI:** 10.1155/2017/1432970

**Published:** 2017-03-29

**Authors:** Ganesan Vadivelan, Pamidighantam Prabhakara Rao, Govindarajulu Venkateswaran

**Affiliations:** ^1^Microbiology & Fermentation Technology Department, CSIR-Central Food Technological Research Institute, Mysore, India; ^2^Resource Centre, CSIR-Central Food Technological Research Institute, Habsiguda, Hyderabad, India

## Abstract

Objectives of this study were designed for improved production of mycelial omega-3 fatty acids with particular reference to EPA and DHA from the oleaginous fungus* Mortierella alpina* CFR-GV15 under submerged low temperatures fermentation supplemented with linseed oil and garden cress oil as an additional energy source. The fungus was grown at 20°C temperature for four days initially followed by 12°C temperature for next five days. The basal medium contained starch, yeast extract, and a blend of linseed oil (LSO) and garden cress oil (GCO) in the ratio 1 : 1. Results of the study revealed that, after nine days of total incubation period, the enhancement of biomass was up to 16.7 g/L dry weight with a total lipid content of 55.4% (v/w). Enrichment of omega-3 fatty acids indicated a significant increase in fatty acid bioconversion (ALA 32.2 ± 0.42%, EPA 7.9 ± 0.1%, and DHA 4.09 ± 0.2%) by 2.5-fold. The two-stage temperature cultivation alters the fatty acid profile due to activation of the desaturase enzyme in the cellular levels due to which arachidonic acid (AA) content reduced significantly. It can be concluded that* Mortierella alpina* CFR-GV15 is a fungal culture suitable for commercial production of PUFAs with enriched EPA and DHA.

## 1. Introduction

Omega-3 fatty acids, including alpha-linolenic acid, eicosapentaenoic acid, and docosahexaenoic acid, have been proved to be cardioprotective, normal neural development, antidiabetic, hypocholesterolemic, and so forth [1]. Apart from the aforesaid physiological benefits, *ω*-3 PUFAs have also been found to be beneficial in alleviating psychosomatic disorders like mood, depression, bipolar disorder, schizophrenia, and dementia [2]. Zygomycetes fungi* Mortierella* sp. has been identified as a source of PUFAs mainly arachidonic acid (AA) at an average value of 30–70% and other PUFAs such as linoleic acid (LA), gamma-linolenic acid (GLA), and dihomo-gamma-linolenic acid (DHGLA) at significant levels [3]. Intake of a diet containing more arachidonic acid may result in changes of selectively increased inflammation or inflammatory responses in humans [4]. However, it has been observed that* M. alpina* could accumulate fatty acids of chain length C:20 including EPA and DHA [5]. Hence,* M. alpina* could be exploited for accumulation omega-3 PUFAs by the activation of desaturase enzymes and also by altering the growth conditions such as low temperature incubation at suboptimal levels. Earlier studies reported that when* M. alpina* was incubated at a low temperature of 5°C, a temperature-sensitive desaturase enzyme was activated which catalyzed the formation of EPA from DHGLA [6, 7]. It was also observed that lowering of temperature along with supplementation of vegetable oil as a carbon source triggered a particular fatty acid precursor to form the very long chain polyunsaturated fatty acid (VLC-PUFAs) such as EPA and DHA in the fungal mycelial mass [8]. In another experiment, it was observed that addition of vegetable oils to the culture medium increased cellular lipid with an altered fatty acid composition. Thus it is evident that supplementation of vegetable oils as carbon source affected not only the structure of the lipid but also the length and degree of desaturation of acyl chains [9]. Earlier research works on* Mortierella* were emphasized on submerged cultivation mainly screening for more potent strains, suitable substrates, and cultivation conditions [10–12]. All the previous studies of this fungus were concentrated on variation of cultural parameters for enhanced production of omega-6 fatty acids [13–15]. Vegetable oils containing different fatty acids served as precursors for biosynthesis of long-chain fatty acids (LC-FA) and supplementation with vegetable oil at 1% level in* Mortierella alpina* ATCC 32222 and microalgae* Physcomitrella patens* has been reported to enhance biomass with PUFAs [16, 17]. LSO and GCO have been reported to be rich sources of ALA and may serve as natural precursors for production of very long chain PUFAs. Further, GCO is highly stable oil possessing high concentrations of natural antioxidants such as the tocopherols and carotenoids [18]. The present work was designed to understand the effect of the blend oil supplementation LSO and GCO on* M. alpina* lipid and its fatty acid composition. Blend oil supplementation (LSO and GCO) on* M. alpina* lipid at two-stage low temperature incubations is being reported for the first time for bioconversion of precursor fatty acids to produce enhanced EPA and DHA accumulation by triggering the activity of Δ^5,6,17^ desaturase enzymes in the mycelial cells of* M. alpina*.

## 2. Materials and Methods

### 2.1. Microorganisms and Maintenance

A novel strain of* M. alpina* CFR-GV15, an arachidonic acid accumulating oleaginous fungus, was screened from the soil samples of Western Ghats of India. The geographical coordinates of the isolation site are latitude N 12°45′14.2351′′ and longitude E 75°38′38.7031.

### 2.2. Nucleotide, BLAST, and Phylogenetic Analysis

The* M. alpina* CFR-GV15 was cultivated in SYM medium at different temperatures: (a) 28°C for seven days, (b) 20°C for seven days, and (c) 20°C for four days followed by 12°C for five days [6, 7]. Genomic DNA was extracted and the nucleotide sequence was analyzed by BLAST and deposited in NCBI [19]. Further the omega-3 desaturase gene was subjected to polymerase chain reaction. A set of degenerate primers were designed that were derived from conserved regions within the gene cluster of five PUFA producing microorganisms. Genomic DNA of* M. alpina* CFR-GV15 was used as a template for PCR with amplification. The PCR was carried out using a thermocycler (Eppendorf, USA), starting at 50.7°C for 2 min and at 54.7°C for 1 min, followed by 30 cycles of 1 min at 7°C and the 10 min hot-start at 95.7°C. The amplified PCR product was purified with QIA quick, and final extension of 10 min at 72.7°C PCR purification columns (Qiagen, Germany), following the manufacturers' protocol. The purified 18S rRNA gene PCR product was directly subjected to DNA sequencing using gene-specific PCR primers. The nucleotide sequence was analyzed by BLAST [20] and the gene sequences determined were submitted to GenBank under the accession numbers KU517437 omega-3 desaturase genes. The partial gene sequences obtained were aligned by BLAST and a phylogenetic tree was constructed by using a software Phylogeny.fr [21]. The omega-3 desaturase gene sequences obtained from NCBI were AB118663 (Lachancea kluyveri SK-FAD3), AB182163.1* (Mortierella alpina)*, KF433065 (*Mortierella alpina* ATCC32222), HQ612176* (Mortierella alpina)*, and AY373823.1* (Saprolegnia diclina)*. The neighbor-joining method with bootstrap of 500 replicates generated the phylogenetic tree. The sequences of the BLAST hits showing significant homological were aligned by ClustalW program [22]; protein sequences were used for generating the genomic123.com software. Comparison of deduced amino acid sequences of* M. alpina* CFR-GV15 desaturase with that of related expression with low to higher temperature was carried out.

### 2.3. Blend Oil Preparation

Oilseeds, namely, mustard, rice bran, soya bean, sunflower, linseed, and garden cress seed, were purchased from the local market. The seeds were powdered and extracted using a Soxhlet apparatus for recovering the fixed oil using hexane (Rendell Tech, VELP Scientifica, Italy). The different combinations of oil blends of “SFO,” “RBO,” “SBO,” “MO,” “GCO,” and “CO” were prepared individually in the ratio of 1 : 1. The physical blending of edible oils was carried out by a reported method [23] by continuously mixing the mixture for 12 h at room temperature.

### 2.4. Cultivation

The seed culture was prepared in 50 mL medium containing (g/L) glucose, 20 g, and yeast extract, 10 g; the pH was adjusted to 6.0 and culture was incubated for 48 h at 28 ± 0.2°C. The inoculum culture was prepared by growing cells in starch yeast extract medium (SYM) and the composition was as follows: (g/L) starch, 10 g; yeast extract, 5 g; KNO_3_, 10 g; KH_2_PO_4_, 1 g; and MgSO_4_, 0.5 g. Crude extracted oils of mustard (MO), rice bran (RBO), soya bean (SBO), sunflower (SFO), linseed (LSO), and garden cress seed (GCO) were incorporated in SYM cultivation medium at 2% level as the additional carbon source. Alpha-linolenic acid (ALA) rich edible oils such as LSO : GCO were added in the ration of 1 : 1. The pH was adjusted to 6.5 before autoclaving at 121°C for 15 min. The cultures were incubated at 20°C and maintained for four days and shifted to a stress condition of 12°C for further five days. The cultures were continuously kept on a rotary shaker (210 rpm) under submerged conditions for the total period of nine days.

### 2.5. Harvesting and Dry Biomass Determination and Lipid Extraction

The biomass was harvested after nine days of incubation and filtered with a wet muslin cloth and the externally adhering surface binding oil was removed by washing off extensively with distilled water three times followed by Tween-80 and rapidly washing with chloroform. The biomass was free frozen by keeping at −80°C initially for 4 h and further freeze-dried for 6–8 h (Labconco Free Zone; Kansas City MO; USA). The freeze-dried biomass was ground into a fine powder and placed in thimbles of dimension 33 mm × 80 mm and the lipid was extracted using a Soxhlet apparatus (Rendell Tech, VELP Scientifica, Italy) using hexane as the solvent.

### 2.6. Analytical Methods and FAME Preparation

Fatty acid methyl esters (FAMEs) of freshly obtained microbial oil of* M. alpina* CFR-GV15 were prepared using 20–40 mg sample employing the method of Morrison and Smith [24] with a minor modification. Methanolic KOH (1 mL) was used for saponification and 1 mL of 14% BF_3_ (Sigma-Aldrich, USA) as the methylating agent. The mixture was incubated at 65°C for 1 h. The derivatized lipids were extracted into hexane and the solvent layer was added with 1 mL of water and centrifuged at 8944 ×g for 5 min. The hexane layer was evaporated under a stream of N_2_ and dissolved in 1 mL of benzene and unwanted solids were again removed by centrifugation at 8944 ×g for 3 min. Gas chromatography analysis of FAMEs was carried out using a RTX-2330 (fused silica) 30 m capillary column of 0.25 *μ*m internal diameter (Shimadzu 2014, Japan). The column temperature was programmed as 160°C (initial) and raising the temperature at the rate of 5°C/min to 250°C with a holding time of 10 min. Carrier gas was nitrogen with total flow rate of 49.5 mL/min. The injector and detector temperature were 240°C and 250°C, respectively. The fatty acids were identified by comparison with standard reference (C4–C24) fatty acid methyl esters (FAMEs) (Sigma-Aldrich, St. Louis, MO).

### 2.7. Statistical Analysis

All experiments were conducted in triplicate and data on experimental microbial biomass and PUFAs production was subjected to analysis of variance and Duncan's multiple range test (*P* ≤ 0.05) using SPSS 16.0 software.

## 3. Results

In our earlier studies,* M. alpina* CFR-GV15 cultivated in glucose, yeast extract basal medium, and pH 6 to 6.5 and incubated at 28 ± 0.2°C for 7 days produced a maximum lipid content of 44.3 ± 0.3% of biomass on dry basis. GC analysis of total lipid revealed the presence of EPA (3.40 ± 0.1%), DHA (4.32 ± 0.1%), and n-6 PUFAs AA (56.7 ± 0.2%) [19]. A significantly higher accumulation of AA was achieved within seven days of fermentation. In the present experiment, it was observed that* M. alpina* CFR-GV15 when grown at 20°C for four days produced a considerable amount of AA and when continued at reduced temperature of 12°C for another five days resulted in the enhancement of EPA and DHA contents and considerably lowered levels of AA. The enhancement of PUFA can be attributed to the trigger of Δ^5,6,17^ desaturase enzyme activity inside the cell. Further the morphology of this fungus in the growth medium at 20°C during the lag phase was spherical, in the early log phase between 72 and 96 h semipellet form, and in the log phase between 96 and 216 h huge pulpy structure when the culture was shifted to a low temperature of 12°C. Similarly, the pH of the cultivation medium initially showed 6.5, gradually increased to 7.1 at 96 h of the growth period and 5.4 at 216 h. It was also observed that fatty acid profile of the biomass varied with time of incubation period. Maximum amounts of GLA, DGLA, and AA were observed after 96 h at 20°C and higher EPA and DHA after 216 h at 12°C. Fatty acid profiles of the different monounsaturated fatty acid rich vegetable oil such as SFO, SBO, RBO, and MO and ALA-rich edible oils LSO, GCO, and CO are presented in Table 1. Fatty acid profile various oil blends are presented in Table 2. The impact of the individual oil supplementation into the medium on omega-3 fatty acid composition of biomass was critically analyzed, and the results are indicated in Table 3. Culture medium supplemented with PUFA rich individual edible oil showed increased biomass with their major fatty acid in the cell biomass. LSO gave the highest biomass followed by CO supplementation. It was also noticed that culture medium supplemented with general vegetable oils gave the low yield of biomass, lipid, and individual PUFA levels when compared to ALA-rich oils such as GCO, CO, and LSO, when LSO supplement in the growth medium could be able to convert the DHGLA into EPA by the action of Δ^5,6^ desaturase enzymes and similarly LSO supplementation resulted in the highest yield of DHA followed by CO and GCO supplementations. This could be due to the activation of Δ^17^ desaturase enzyme in the cells. The levels of EDA, ETA, and AA were reduced during this process which can be attributed to metabolic conversion in the fatty acid pathway. LSO was blended with other vegetable and edible oils to establish the metabolic alteration for improved fatty acids production especially the omega-3 fatty acids. The major combination of edible oil such as LSO-GCO; LSO-CO; and CO-GCO showed significantly (*P* ≤ 0.05) increased biomass and *ω*-3 fatty acid; notably the EPA and DHA at end of the cultivation period and the GC profiles revealed that AA content was found to be reduced sharply under these conditions. Similarly, vegetable oil combinations of LSO-SBO; LSO-RBO; LSO-MO; and LSO-SFO showed very less AA conversion (Table 3). However, when LSO-GCO, LSO-Co, and GAO-CO blends were used as supplements of carbon source, reduction in AA was considerable and a complementary increase in EPA and DHA contents was observed. In all the blended oil supplementation experiments ALA was found to remain almost constant with all the blends.

In the experiment, it was found that, during the growth stage of* M. alpina* at 20°C with LSO : GCO as blended oil supplementation, maximum amounts of AA (7.3 ± 0.0) and ALA (46.2 ± 1.3) were observed on the fourth day which subsequently decreased to 2.9 ± 0.2 and 32.2 ± 0.7, respectively, after the 9th day with lowering of temperature from 20°C to 12°C (Table 4). The increasing trend for EPA (20:5, n-3) and DHA (22:6, n-3) during the same period was observed from 2.8 ± 0.2 to 7.9 ± 0.1 and 0.6 ± 0.0 to 4.09 ± 0.2, respectively (Table 4).

**Figure 1:**
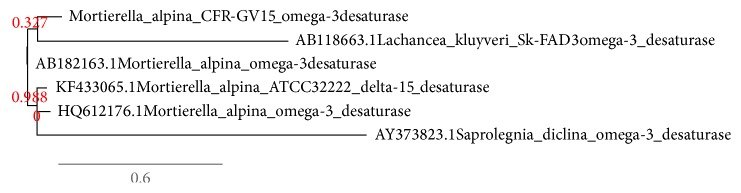
Phylogenetic analysis of the omega-3 desaturase gene diversity between the long chain EPA and DHA producers.

**Figure 2:**
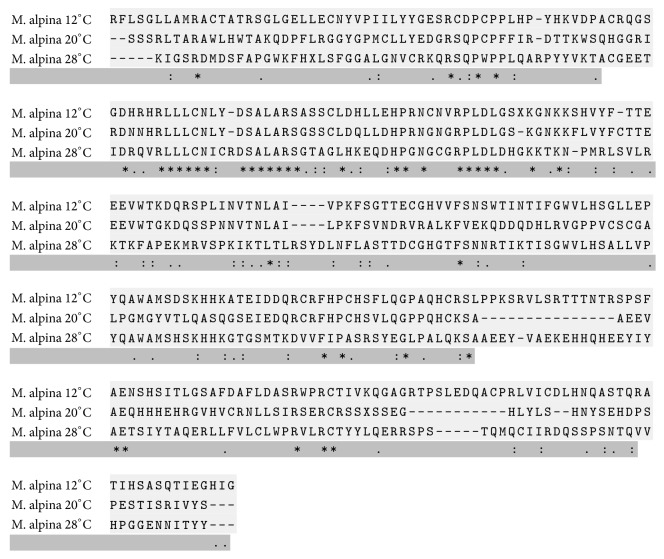
Multiple sequence alignment of* Mortierella alpina* CFR-GV15 different temperature depending on gene expression of omega-3 desaturase. The deduced amino acid sequence of* M. alpina* omega-3 desaturase was subjected to BLAST analysis, after which the closest relatives of gene expression were selected and aligned using ClustalW.

The fungal strain of* M. alpina* CFR-GV15 has been isolated and identified using 18S rRNA. The fungal sequences were compared with known 18S rRNA sequences from GenBank and EMBL using the BLAST search engine (https://blast.ncbi.nlm.nih.gov/Blast.cgi). The deduced amino acid sequence of* M. alpina* omega-3 desaturase was subjected to BLAST analysis (Figure 1). Further, the closest relative gene expression was selected and aligned using ClustalW (Figure 2).

## 4. Discussion

*Mortierella* sp. has been found to produce the high concentrations of PUFA depending upon the fermentation media, C/N ratio, pH, and temperature. In the present experiment* M. alpina* grown at two-stage shifting temperatures, namely, 20°C and 12°C, respectively, for nine days produced lipids with higher EPA and DHA content by triggering the Δ^5^, Δ^6^, and Δ^17^ desaturases in the cell. The reduction in ALA and increase in EPA and DHA contents in the fatty acid profile clearly indicate that the desaturase enzyme is involved in conversion. Several studies have clearly shown that Δ^5^ desaturase, the enzyme that catalyzes the conversion of DGLA to AA, could be inhibited by the presence of certain oils containing PUFAs in the growth media [10, 25, 26]. The composition of SYM medium was slightly altered by replacing glucose with potato starch, certain vegetable oils, and combination of oils as additional carbon sources. Jang et al. [16] and Nisha and Venkateswaran [10] reported that pH 6.5 was optimum to produce more arachidonic acid in the mycelial cells of* M. alpina* in five to seven days of incubation. Parameters were similar in the present experiments; however the culture was incubated at two temperature conditions. Another study indicated that this strain effectively utilized simple sugars for the growth and production of polyunsaturated fatty acids [10, 27]. The significant fact is that the utilization of oils by microorganisms is accompanied by the production of extracellular lipases, which cleave fatty acid residues from glycerol. Moreover, the fatty acids production can either be incorporated into lipid structures or degraded to basic skeletons serving the biomass synthesis [10, 28]. Similar observations were also made by Akhtar, Koike et al., Ratledge, Hao et al., and Hao et al. [11, 15, 28–30]. Our earlier report indicated that different sources of N_2_ supplementation in the growth medium altered the fatty acid profile in* Mucor rouxii* CFR-G15 using [11] RSM and* Mortierella alpina* CBS 528.72 considerably. The study also indicated that the combination of both organic and inorganic nitrogen sources gave the highest cell biomass [10, 31]. In the present experiment, culture medium was incorporated with organic and inorganic N_2_ and wide ranging changes were observed in the fatty acid profiles of lipids. The changes observed in the pH may be due to the utilization of nitrogenous sources from the growth medium. Thus, we can conclude that carbon, nitrogen sources, oil supplementation, and temperature play an essential role in converting PUFAs into desired fatty acids such as EPA and DHA in* M. alpina* CFR-GV15 strain. All these observations are concomitant with the study of Shinmen et al. [32], wherein they reported that the addition of oils allowed cell biomass to convert C18 fatty acids substrates into LC-PUFAs. MO, RBO, and SFO supplementation in the medium increased the accumulation of AA and stimulates cell growth and bioconversion of the AA into EPA and DHA by lipogenic pathway at the cellular level [33] and supplementation of oils stimulates cell growth and formation of PUFAs production in* M. alpina* ATCC 32222 [16]. The percentage of AA before converting EPA and DHA was 56.7% which reduced to 6.95% on the seventh day of incubation, 5.2% on the eighth day, and 2.9% on the ninth day (Figure 2). Similarly, efficiency of ALA conversion in SYM medium with blended oil was also shown to be 40% on the seventh day at 12°C which reduced to 34.3% on 8th day and 32.5% on the ninth day. This can be attributed to higher degree of activation of the Δ^17^ desaturase enzyme at the lower temperature and subsequent bioconversion of DGLA to ETA and AA to EPA and DHA (Table 5). A rare phenomenon was observed in this strain when LSO : CO blend was supplemented in the growth medium and incubated at low temperature, a conversion of EDA to DHA, a higher degree of activation of desaturase enzyme had occurred (data not shown). LSO : GCO 1 : 1 blended oil during fermentation showed a significant increase in fatty acid conversion (ALA 32.2 ± 0.42%, EPA 7.9 ± 0.1%, and DHA 4.09 ± 0.2%) and intermediate product was also observed. Several studies [13, 32, 34] have revealed that the mycelial EPA content can be increased by the cultivation of the fungi with linseed oil that contains a high proportion of alpha linolenic acid (ALA). Similarly, the strain* M. alpina* also exhibits higher EPA production with the addition of ALA-containing single oils, such as LSO oil 3% in the medium at 12°C, where EPA accumulation reached 66.6 mg/g dry mycelia [34]. Basal medium containing soluble starch as the carbon source combined with linseed oil in 2 : 1 ratio for 7 days' incubation produced 3.56% EPA and 0.18% DHA [33].* M. alpina* 1S-4 produced EPA (approximately 10% of total fatty acids) when the growth temperature was below 20°C [25]. In another report,* M. alpina* ATCC 32222 vegetable oil supplementation with 1% linseed oil or sunflower oil individually at 20°C for three days followed by 12°C for five days resulted in a maximum yield of EPA (3.56 ± 0.23%) and DHA (0.23 ± 0.03%) [16]. By using a defective mutant of desaturase, EPA-rich oil with a low level of AA was obtained; Mut48 converted exogenous ALA to EPA [35].

## 5. Conclusion

*M. alpina* CFR-GV15, a novel isolate from the soils of Western Ghats of India, produced 16.7 g/L dry weight basis (DW) biomass and 55.4% total lipid when shifted to low temperature in the presence of LSO : GCO blended oil at 2% level in the fermentation medium. The conversion of *ω*-6 fatty acids such as GLA, DHGLA, and AA to *ω*-3 fatty acid, especially the EPA and DHA, was possible by the change in the pathway utilizing elongase as well as Δ^6,5,17^ desaturase enzymes. The present study also revealed that* M. alpina* CFR-GV15 showed a significantly increased VLC-PUFAs accumulation when supplemented with blended oil compared to single oil supplementation. The increasing applications of EPA and DHA and its limited conventional sources have led to an extensive search for alternative production. In the recent past, renewable sources of EPA and DHA from genetically modified algae, yeast, fungi, or other microorganisms are becoming more popular. Further, microorganism-based processes could be reliable, economical, and attractive for large-scale production of EPA and DHA.

## Figures and Tables

**Table 1:** Fatty acid composition of edible and vegetable oils.

Fatty acid (%)	LSO	GCO	CO	MO	RBO	SBO	SFO
C14:0	0.6 ± 0.2	0.4 ± 0.2	ND	ND	0.4	ND	ND
C16:0	15.6 ± 0.1	11.2 ± 0.3	12.3 ± 0.0	2.7 ± 0.0	23.4 ± 1.1	16.3 ± 1.3	6.4 ± 0.3
C18:0	2.1 ± 0.4	2.6 ± 0.8	3.4 ± 0.1	1.3 ± 0.3	2.1 ± 0.2	4.3 ± 1.1	3.2 ± 0.0
C18:1	18.4 ± 2.2	22.7 ± 1.2	14.3 ± 0.8	13.2 ± 0.7	42.2 ± 2.0	26.4 ± 1.4	44.2 ± 0.7
C18:2	6.3 ± 2.1	11.9 ± 3.1	8.5 ± 0.2	14.7 ± 0.2	30.5 ± 0.2	49.9 ± 2.1	46.2 ± 2.2
C18:3	56.4 ± 0.3	34.2 ± 1.0	61.3 ± 0.5	16.5 ± 0.1	1.4 ± 0.7	3.3 ± 0.3	ND
C20:0	0.4 ± 0.8	13.2 ± 0.3	0.3 ± 0.2	1.7 ± 1.5	ND	ND	ND
C21:0	0.22 ± 1.7	3.8 ± 0.0	0.23 ± 0.1	ND	ND	ND	ND
C22:1	ND	ND	ND	50.2 ± 1.3	ND	ND	ND
SFA%	17.7 ± 2.6	13.8 ± 1.1	15.7 ± 0.1	4.0 ± 0.3	25.5 ± 1.3	20.6 ± 2.4	9.6 ± 0.3
MUFA%	18.4 ± 2.2	22.7 ± 1.2	20.3 ± 0.8	13.2 ± 0.7	42.2 ± 2.0	26.4 ± 1.4	44.2 ± 0.7
PUFA%	62.7 ± 2.4	46.1 ± 4.1	69.8 ± 0.7	31.2 ± 0.3	31.9 ± 0.9	53.2 ± 2.4	46.2 ± 2.2

LSO: linseed oil, GCO: garden cress oil, CO: chia oil, SFO: sunflower oil, RBO: rice bran oil, MO: mustard oil, SFO: sunflower oil, SFA: saturated fatty acids, MUFA: monounsaturated fatty acids, PUFA: polyunsaturated fatty acids, and ND: not detected.

**Table 2:** Fatty acid composition of various oil blends.

Fatty acid (%)	LSO : SBO	LSO : RBO	LSO : MO	LSO : SFO	LSO : GCO	LSO : CO	CO : GCO
C14:0	0.32 ± 0.6	0.4 ± 0.2	0.2 ± 0.0	0.18 ± 0.3	0.2 ± 0.1	0.17 ± 0.0	0.11 ± 0.1
C16:0	6.1 ± 2.1	8.4 ± 0.1	0.2 ± 0.7	2.1 ± 0.2	7.3 ± 0.3	6.8 ± 1.0	6.8 ± 0.5
C18:0	4.3 ± 1.1	2.1 ± 0.2	1.3 ± 0.3	3.2 ± 0.0	3.6 ± 1.1	3.8 ± 0.2	4.4 ± 0.2
C18:1	26.4 ± 1.4	38.2 ± 1.0	13.2 ± 0.7	31.2 ± 0.7	21.9 ± 2.1	16.4 ± 0.7	17.8 ± 1.1
C18:2	29.3 ± 2.1	24.3 ± 1.2	19.7 ± 1.9	37.7 ± 1.8	12.1 ± 0.8	14.7 ± 0.3	14.9 ± 0.7
C18:3	27.3 ± 0.3	21.4 ± 0.7	26.5 ± 0.1	23.2 ± 1.2	44.7 ± 1.7	47.3 ± 2.1	51.3 ± 2.3
C20:0	0.3 ± 0.0	0.23 ± 0.4	1.2 ± 0.5	0.22 ± 0.2	0.8 ± 0.0	2.6 ± 0.2	0.4 ± 0.0
C21:0	2.2 ± 0.2	2.1 ± 0.1	35.2 ± 1.3	0.5 ± 0.1	3.6 ± 0.1	1.5 ± 0.0	2.14 ± 0.0
C20:4	3.1 ± 0.4	2.2 ± 0.3	1.3 ± 0.6	1.8 ± 0.3	3.1 ± 0.2	1.3 ± 1.0	2.0 ± 0.0
Others	0.9 ± 0.6	1.2 ± 1.2	1.3 ± 0.1	0.7 ± 0.2	2.3 ± 1.4	4.7 ± 2.1	0.2 ± 0.3
SFA%	10.4 ± 3.2	10.5 ± 0.3	1.5 ± 1.0	5.3 ± 0.2	10.9 ± 1.4	10.6 ± 1.2	11.2 ± 1.3
MUFA%	26.4 ± 1.4	38.2 ± 1.0	13.2 ± 0.7	31.2 ± 0.7	21.9 ± 2.1	16.4 ± 0.7	17.8 ± 1.1
PUFA%	59.7 ± 2.8	47.9 ± 2.2	46.2 ± 2.6	62.7 ± 3.3	56.9 ± 2.7	61.3 ± 2.4	68.2 ± 3.0

LSO: linseed oil, GCO: garden cress oil, CO: chia oil, SFO: sunflower oil, RBO: rice bran oil, MO: mustard oil, SFO: sunflower oil, SFA: saturated fatty acids, MUFA: monounsaturated fatty acids, PUFA: polyunsaturated fatty acids, and ND: not detected.

**Table 3:** Effect of individual vegetable and blended oil supplementation as carbon source on *M. alpina* CFR-GV15 on biomass, total lipid, and omega-3 fatty acid production.

Oil supplement	Dry biomass (g/L)	Total lipid (% w/w)	Fatty acid content (% w/w)			
			18:3 (n-3)	20:4 (n-6)	20:5 (n-3)	22:6 (n-3)
GY medium	10.8 ± 0.1^c^	44.3 ± 0.3^b^	0.02 ± 0.0	56.7 ± 0.2^a^	3.4 ± 0.1^b^	4.3 ± 0.1^a^
MO	6.3 ± 0.1^f^	32.4 ± 0.9^f^	ND	37.6 ± 0.2^b^	0.7 ± 0.1^e^	0.4 ± 0.1^c^
RBO	7.0 ± 0.0^e^	34.4 ± 0.8^e^	ND	32.6 ± 0.8^c^	1.1 ± 0.1^e^	0.4 ± 0.2^b^
SBO	4.5 ± 0.2^g^	26.5 ± 0.2^g^	ND	26.1 ± 0.3^d^	2.8 ± 0.3^c^	0.1 ± 0.1^c^
SFO	7.3 ± 0.1^e^	32.7 ± 0.3^f^	ND	25.1 ± 0.0^d^	2.4 ± 0.0^d^	0.3 ± 0.1^c^
GCO	8.6 ± 0.1^d^	37.8 ± 0.0^d^	34.2 ± 0.0	5.5 ± 0.7^e^	2.2 ± 0.1^d^	0.5 ± 0.0^b^
CO	11.8 ± 0.2^b^	42.5 ± 0.3^c^	57.9 ± 0.0	8.2 ± 0.5^f^	3.4 ± 0.1^b^	0.2 ± 0.8^c^
LSO	13.3 ± 0.2^a^	49.8 ± 0.4^a^	55.1 ± 0.3	4.0 ± 0.8^e^	5.6 ± 0.2^a^	0.7 ± 0.1^b^
Blend oil supplement (2% level)						
LSO + SFO	9.7 ± 0.2^c^	45.1 ± 0.2^c^	38.2 ± 0.3^d^	26.5 ± 0.1^b^	1.0 ± 0.0^e^	1.0 ± 0.1^d^
LSO + SBO	7.5 ± 0.2^d^	44.2 ± 0.1^d^	39.6 ± 0.4^d^	26.2 ± 0.1^b^	1.5 ± 0.1^d^	0.1 ± 0.8^e^
LSO + RBO	6.6 ± 0.2^e^	40.0 ± 0.4^e^	43.1 ± 0.2^b^	22.1 ± 0.1^c^	1.6 ± 0.3^d^	0.4 ± 0.1^e^
LSO + MO	7.5 ± 0.1^d^	36.8 ± 0.4^f^	41.7 ± 0.1^c^	28.5 ± 0.4^a^	0.7 ± 0.4^f^	0.5 ± 0.3^e^
LSO + GCO	16.7 ± 0.2^a^	55.4 ± 0.3^a^	32.0 ± 0.4^e^	2.84 ± 0.1^f^	7.9 ± 0.1^a^	3.8 ± 0.0^b^
LSO + CO	15.3 ± 0.2^b^	48.2 ± 0.3^b^	40.4 ± 0.7^c^	6.02 ± 0.1^e^	3.4 ± 0.2^c^	4.9 ± 0.3^a^
GCO + CO	15.1 ± 0.5^b^	46.7 ± 0.3^b^	47.6 ± 0.9^a^	8.7 ± 0.1^d^	4.3 ± 0.9^b^	1.3 ± 0.0^c^

Culture conditions: GY medium: glucose and yeast extract 2 : 1 ratio; pH 6.5; 230 rpm; cultivation period 7-day values of means ± SD (control). Optimized medium carbon source: 1% carbon and 0.5% yeast extract; 1% KNO_3_; 0.1% KH_2_PO_4_ and MgSO_4_·7H_2_O 0.05%; additional 2% individual vegetable and blended oil supplementation; pH 6.5; 230 rpm; cultivation period: 9-day values of means ± SD, n-3. Values in the same column that do not share the same alphabetic superscripts are significantly different at levels according to Duncan's multiple range tests.

**Table 4:** Effect of selective blend oil LSO : GCO oil and incubation at low temperature after 4th to 9th day of ALA bioconversion efficiency in *M. alpina* CFR-GV15.

Fatty acid (%)	20°C–12°C low temperatures (4–9 days)					
	4th	5th	6th	7th	8th	9th
C14:0	0.4 ± 0.1	0.5 ± 0.0	0.6 ± 0.1	0.6 ± 0.0	0.3 ± 0.3	0.4 ± 0.4
C16:0	9.8 ± 0.4	9.9 ± 0.4	10.4 ± 1.2	10.3 ± 0.7	10.4 ± 3.1	8.7 ± 0.2
C18:0	0.6 ± 1.3	0.7 ± 1.7	0.8 ± 2.5	0.7 ± 1.2	2.5 ± 1.1	5.3 ± 0.0
C18:1	12.4 ± 0.5	13.4 ± 1.1	15.5 ± 0.1	15.3 ± 2.1	18.0 ± 0.1	20.7 ± 1.3
C18:2	12.6 ± 2.1	13.2 ± 0.7	14.1 ± 0.3	12.4 ± 0.4	10.6 ± 0.0	8.6 ± 2.1
C18:3(n-3)	46.2 ± 1.3	44.0 ± 1.3	43.0 ± 1.3	40.0 ± 2.3	34.3 ± 0.1	32.2 ± 0.7
C20:0	4.3 ± 0.4	6.8 ± 1.0	5.9 ± 1.8	5.8 ± 1.0	6.8 ± 0.4	8.7 ± 0.4
C20:1	0.6 ± 0.2	0.3 ± 0.6	0.2 ± 0.6	0.2 ± 0.7	0.8 ± 1.0	1.0 ± 0.5
C20:4(n-6)	7.3 ± 0.0	6.3 ± 0.5	6.2 ± 0.2	6.9 ± 0.5	5.2 ± 0.0	2.9 ± 0.2
C20:5(n-6)	2.4 ± 0.1	2.1 ± 0.2	2.1 ± 0.8	2.3 ± 0.2	2.5 ± 1.1	0.3 ± 0.0
C20:5(n-3)	2.8 ± 0.2	4.3 ± 0.0	4.2 ± 0.1	4.7 ± 0.0	5.9 ± 0.1	7.9 ± 0.1
C22:6(n-3)	0.6 ± 0.0	0.9 ± 0.1	1.4 ± 0.1	1.6 ± 0.1	2.3 ± 0.2	3.8 ± 0.2

LSO: GCO: linseed oil and garden cress seed oil and SYM: starch yeast extract medium. Values are expressed as mean (*n* = 3) ± SD. Values in the same column that do not share the same alphabetic superscripts are significantly different at *P* ≤ 0.05.

**Table 5:** Effect of shifting temperature and blended oil on omega-3 fatty acid production.

Fatty acid (%)	LSO : GCO^a^ Blend oil	Control Without blend oil^b^	Blend oil supplementation with low temperature at 20°C to 12°C^c^
C14:0	0.2 ± 0.1	0.5 ± 0.0	0.4 ± 0.4
C16:0	9.1 ± 0.0	8.4 ± 0.4	8.7 ± 0.2
C18:0	2.7 ± 0.4	5.1 ± 0.3	5.3 ± 0.0
C18:1	8.8 ± 0.3	6.2 ± 0.5	20.7 ± 1.3
C18:2	7.0 ± 0.0	6.7 ± 0.5	8.6 ± 2.1
C18:3(n-3)	53.2 ± 0.2	5.6 ± 0.2	32.2 ± 0.7
C20:0	NA	NA	8.7 ± 0.4
C20:1	5.3 ± 0.0	3.4 ± 0.3	1.0 ± 0.5
C20:4(n-6)	1.2 ± 0.1	56.7 ± 0.2	2.9 ± 0.2
C20:5(n-6)	0.04 ± 0.0	NA	0.3 ± 0.0
C20:5(n-3)	NA	3.4 ± 0.0	7.9 ± 0.1
C22:6(n-3)	NA	4.3 ± 0.6	4.09 ± 0.2

Culture conditions: ^a^LSO : GCO blended oil 2%; 0.5% yeast extract; 1% KNO_3_; 0.1% KH_2_PO_4_ and MgSO_4_·7H_2_O 0.05%; pH 6.5; cultivation period 9 days at 20°C values of means ± SD. ^b^GY medium: glucose and yeast extract 2 : 1 ratio; pH 6.5; 230 rpm; cultivation period 7 days at 20°C values of means ± SD. ^c^SYM: starch-yeast extract medium; pH: 6.5; carbon source: 1% starch and 0.5% yeast extract; 1% KNO_3_; 0.1% KH_2_PO_4_ and MgSO_4_·7H_2_O 0.05%; additional 2% blended oil supplementation; 230 rpm; cultivation period and temperature: four days at 20°C and shifted to 12°C another five days; 9-day values of means ± SD, n-3. Values in the same column that do not share the same alphabetic superscripts are significantly different at levels according to Duncan's multiple range tests.
